# Development of a tibial experimental non-union model in rats

**DOI:** 10.1186/s13018-021-02408-3

**Published:** 2021-04-14

**Authors:** Xue-Qiang Wu, Dong Wang, Yang Liu, Jun-Lin Zhou

**Affiliations:** 1 Department of Orthopedics, Beijing Chaoyang Hospital, Capital Medical University, 8 Gongren Tiyuchang Nanlu, Chaoyang District, Beijing, 100020 China; 2 Department of Handsurgery, Tangshan Second Hospital, Tangshan, 063000 China

**Keywords:** Non-union, Animal model, Bone healing, Periosteum, Tibia, Rat

## Abstract

**Background:**

Many non-union animal models have been developed to explore the problems surrounding fracture healing. However, the existing models are not perfect and cannot satisfy all non-union studies. This study aimed to make a non-union model of the tibia in rats by cauterization of the posterior of 2 mm on both sides of the fracture end after open osteotomy of the tibia and fixing the fractured tibia with a Kirschner wire 0.8 mm in diameter.

**Methods:**

For this study, 96 female adult Sprague-Dawley (SD) rats were used. The rats underwent surgery to produce a tibial open fracture and were fixed with a 0.8-mm diameter Kirschner wire. In 48 of the rats, the periosteum proximal and distal to the fracture end was cauterized.

**Results:**

At 2, 4, 6, and 8 weeks after surgery, radiological and histological analysis showed typical physiological healing in the control group, and the healing rate was 100% at 6 weeks. But the non-union group was characterized by resorption of the fracture ends with few callus formations and no bridging callus formation, and the healing rate was 0% at 8 weeks.

**Conclusions:**

This method represents a reproducible model to create atrophic non-unions. This model provides a new option for studying the basic healing mechanisms and evaluating new therapies for bone regeneration and treatment of non-unions.

## Background

Despite continuous advancements in fracture repair methods and principles in recent years, non-unions, which are mainly divided into hypertrophic non-unions and atrophic non-unions, remain a major clinical problem. A hypertrophic non-union is characterized by a large callus formation and endochondral bone formation at the fracture ends. In contrast, an atrophic non-union is characterized by sparse callus formation and sclerosis of the medullary canal, with only fibrous tissue filling the fracture gap [1]. Although most hypertrophic non-unions can be successfully treated with stable osteosynthesis, the treatment of atrophic non-unions is often difficult and highly challenging [2].

Many factors are known as the cause of fracture non-union, including instability, infection, loss of vascularity, soft tissue interposition, and soft tissue damage [3–8]. Hence, a non-union model is important when studying the formation and treatment of non-unions. Most previous non-union models have been based on large segmental defects of bone at the fracture site [4, 9]. However, these models do not adequately represent the biological situation in which most non-unions develop [10, 11]. In this context, Hietaniemi et al. have reported a femoral non-union model in rats [10]. The model was produced by an open osteotomy with loose Kirschner wire fixation and cauterization of the periosteum. Kokubu et al. reported a high rate of wire migration and fatigue failure in this model, and the authors established a non-union model themselves [11]. Most of the rat models that have been reported were femoral non-union models [10–13]. Because of the thick muscle tissue around the femur, it is difficult to establish a model or do treatment research about fracture healing. In addition, some treatments, like repetitive brief ischemia, cannot be done on these models [14].

To facilitate the establishment of a model and improve fracture healing intervention, we developed specific modifications to the non-union model developed by Hietaniemi et al. by choosing the tibia in rats to make a non-union model. We made a non-union model of the tibia in rats by cauterization of the periosteum of 2 mm on both sides of the fracture end and fixed the fractured tibia with a 0.8-mm diameter Kirschner wire, different from that of the femur.

## Methods

### Animals

PASS 11.0 software was used to calculate the sample size. We referred to the previous literature by using burning periosteum to make bone nonunion rat model and all of them were successful [11, 15, 16]. In addition, there was no report about making model failure. In order to accurately predict the success rate of model, we set 1-βas 0.95 and *α* as 0.01. Ten rats were needed at each point by calculating. As the accidental mortality of rats was set as 20%, we need 12 rats in each time point. We observed four time points so 96 rats were needed.

We used 96 female adult Sprague-Dawley (SD) rats (8 weeks old; mean weight of approximately 210 g; Charles River Laboratories, Beijing, China) in the current study. The rats were divided into two groups: a control group (48 rats) and a non-union group (48 rats), which underwent surgery to produce tibial open fractures and tibial non-unions, as described below. All procedures were performed in accordance with the Guiding Principles in the Care and Use of Animals and approved by the Capital Medical University Committee on the use of animals in research and education. The approval number was AEEI-2020-092. The animals were maintained in a controlled condition of 12 h light and 12 h dark at 23.6 °C, with humidity at 35%, and were fed a standard chow diet with free water intake. At the end of the experiment, the rats were euthanized using pentobarbital sodium.

### Surgical procedures

#### Operative method: non-union

All surgical procedures were performed under normal sterile conditions. After a 1-week acclimation period, the rats were anesthetized with pentobarbital sodium administered intraperitoneally at a dosage of 40 mg/kg of their body weight. The right hind leg of the rat was shaved and disinfected by iodophor after successful anesthesia. A 1.5-cm skin incision was made over the anterior of the tibia in the middle, and blunt dissection was used to separate the skin from the muscle. The tibia muscles were divided longitudinally in the middle third from the tibia, and a transverse osteotomy of the mid diaphysis of the right tibia was made using an oscillating mini saw. At the same time, the fibula was broken. The medullary cavity was entered from the fracture site and reamed proximally and distally. The medullary cavity was washed with a 0.9% saline solution. The periosteum was cauterized circumferentially 2 mm proximally and distally to the fracture site with a loop tip surgical cautery (Dalian Sanmu, China). The cautery tip was bent into a rectangle with a width of 2 mm before the operation. All soft tissue was protected except for the periosteum around the fracture site. A 0.8-mm steel Kirschner wire (Zimmer, USA) was used to open the cortical bone and medullary canal by touching the tibial plateau border when the knee was bent at a 90° angle. The skin was stabbed directly by the wire, and the wire was driven into the proximal medullary canal of the fractured tibia. After reduction, the implant was inserted distally through the medullary canal up to the distal part, and the tibia fracture was fixed with the 0.8-mm steel Kirschner wire. The protruding part of the wire was cut flush with the cortical level of the bone. The fracture fixation was completed manually. The incision was closed in layers with 5-0 unabsorbable sutures.

#### Operative method: control

The operative legs of the rats were prepared in the same manner as the non-union group. A midshaft tibial fracture was created the same way as in the non-union group. The tibial shaft was then rodded using the technique as described for the non-union group. The periosteum was not treated in this group.

### Experimental protocol

Unprotected weight-bearing was allowed immediately for both groups. After stabilization, X-ray examinations were performed to document the fracture reduction and the position of the implant. Six rats with comminuted fractures in radiographs were excluded from the study and replaced with other animals. There were no infections in our experiment.

Each group had 48 animals assigned. They were maintained for intervals of 2, 4, 6, or 8 weeks. After a radiographic examination, eight specimens from each time point were randomly selected for micro-CT evaluation. The four remaining specimens from each group were processed for histological study.

### Radiographic evaluation

Radiographs were taken throughout the observation period at days zero (days after fracture), 2 weeks, 4 weeks, 6 weeks, and 8 weeks. Digital X-rays were taken using an animal X-ray unit (Avchoice, Del, USA). This was done under anesthesia with the animal prone and both limbs fully abducted. They were taken in two directions: anteroposterior and lateral views. Each view had two cortical gaps. A fracture union was defined as a bridging callus on more than three cortices of four cortical gaps in two directions. Two blinded observers assessed the radiographs of each animal to judge whether the fractures united or not.

We used micro-CT (Bruker Skyscan1176, Belgium) to inspect the bone healing at 2, 4, 6, and 8 weeks. The rats were euthanized, and their lower limbs were harvested. The Kirschner wires were taken out before the micro-CT examination. Source voltage was set to 65 kV, and the current was 381 uA. The image pixel size was 18 μm, and the filter used was Al 1 mm. The rotation step was at 0.5°, and a 180° rotation was used. An NRecon 1.6.10.2 (Bruker, Belgium) made a two-dimensional reconstruction, and the region of interest (ROI) was 250 axial slices above and below the fracture line. Sequential images were obtained at pixel sizes of 18 μm and slice distances of 18 μm. The resulting image width and height were 2000 pixels. Three-dimensional reconstruction of 500 tomograms was performed using the computer software (CTVox).

### Histological evaluation

At the end of the maintenance intervals, the 32 rats utilized for histological evaluation were euthanized by excessive intraperitoneal pentobarbital. The right tibias were harvested, and bone tissue was fixed in 4% polyformaldehyde for 24 h at 4 °C, defatted in ethanol, and decalcified with 5% EDTA at 4 °C. Then, the samples were dehydrated in alcohols and embedded in paraffin. Paraffin sections were cut at 4 μm in thickness along the longitudinal axis. These sections were stained with hematoxylin and eosin for histological observation. The histology was evaluated to confirm that the non-union group model produced an established non-union and that the control group model formed a bridging callus.

The animals did not experience pain during our study.

### Statistical analysis

SPSS 23.0 was used to analyze the data. Measurement data and enumeration data were reported as median (interquartile) and percentage, respectively. We used Kruskal-Wallis *H* test to compare multiple groups and Mann-Whitney *U* test to compare two groups as measurement data. Enumeration data were analyzed by *χ*2 test or Fisher definite probability methods. *P*<0.05 was considered as statistically significant.

## Results

### Digital X-rays

**Fig. 1:**
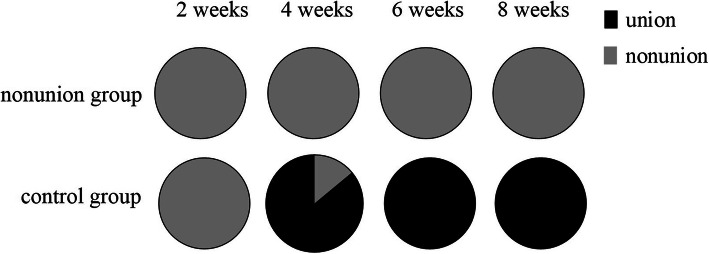
The pie graph for each group. The incidence rate of bone union in non-union group was zero at 2, 4, 6, and 8 weeks. All rats in the control group have fractures healed at 4 weeks

Radiographs were taken after the operation was finished. Transverse midshaft tibial fractures were shown in both the non-union group models and the control group models. No Kirschner wire bending, breaking, or displacement was observed throughout the experiment period. The rates of fracture unions of each group during each period are shown in Fig. 1.

**Fig. 2:**
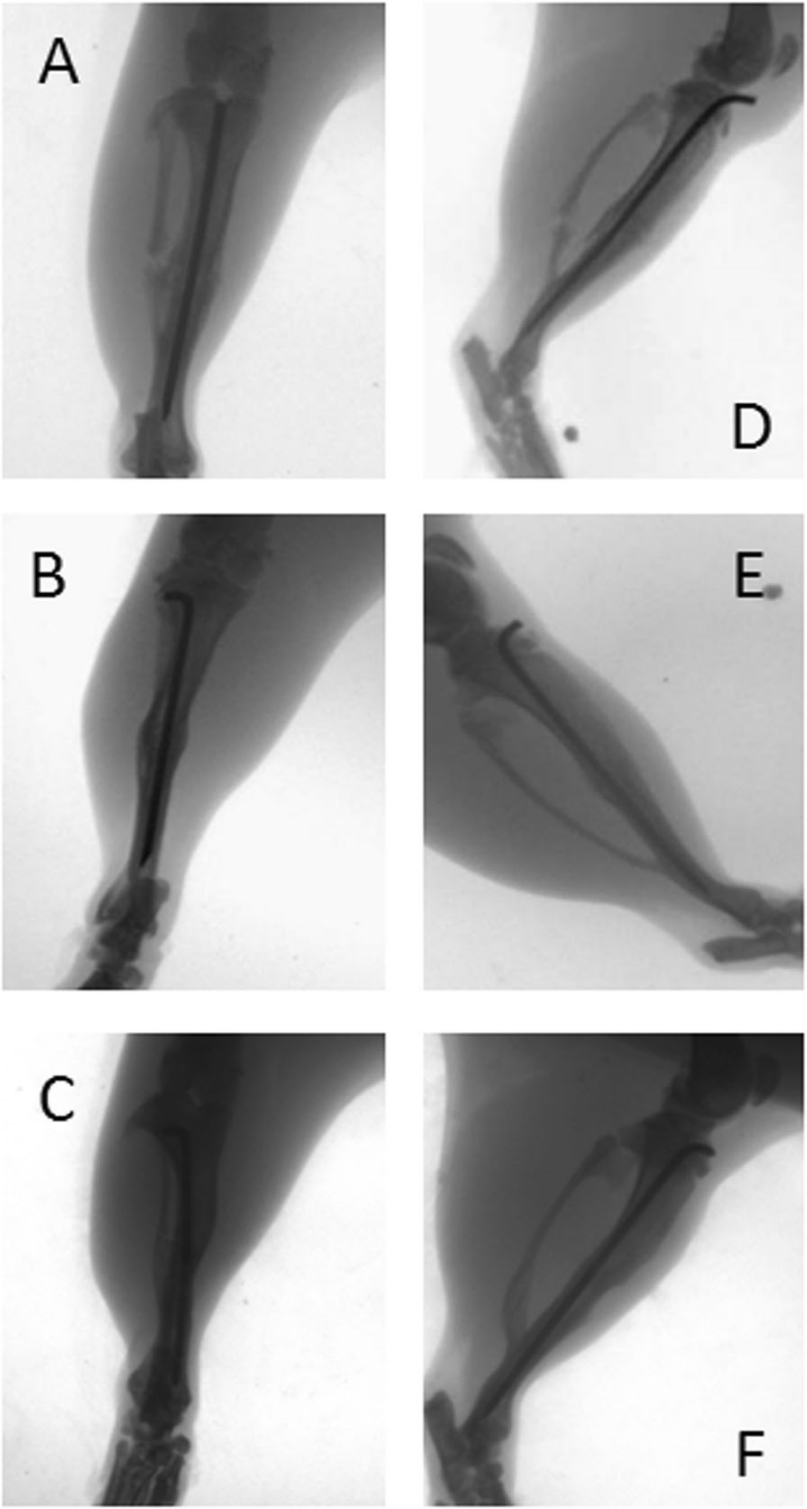
The anteroposterior and lateral view of the X-rays of the control group. The radiographs were obtained at 2 weeks (**a** and **d**), 4 weeks (**b** and **e**), and 6 weeks (**c** and **f**) after surgery. It can be seen that the fracture healed well at 4 and 6 weeks

**Fig. 3:**
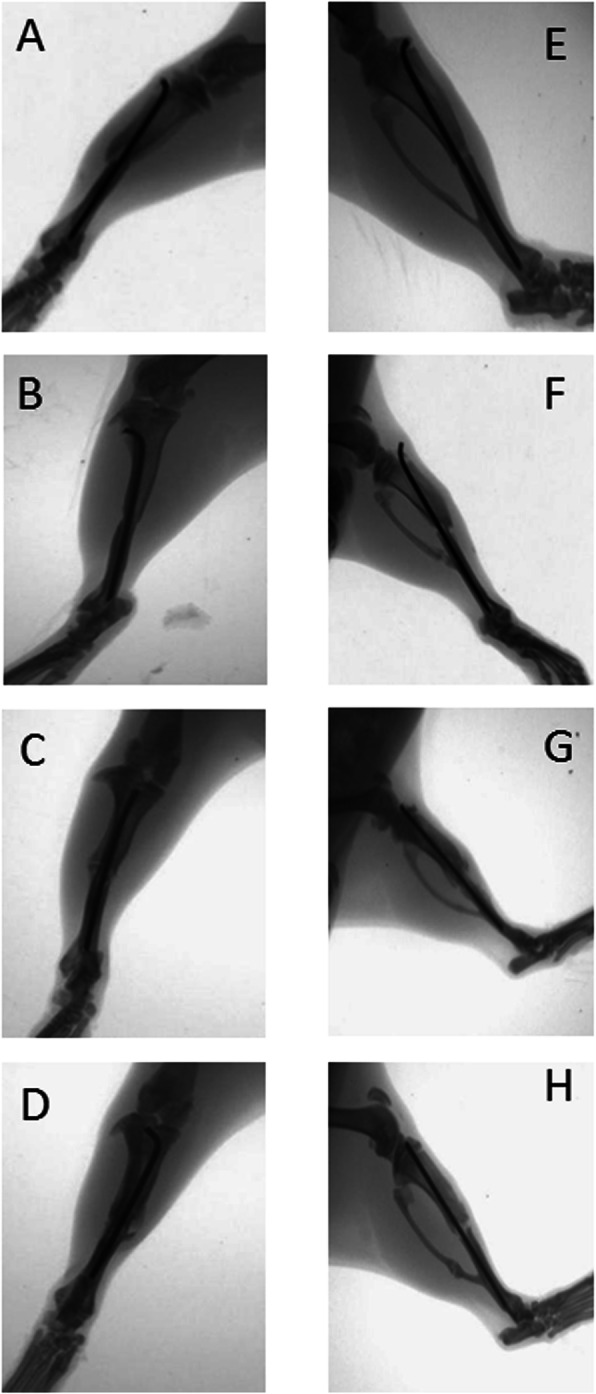
X-rays of the rats in the non-union group, including the anteroposterior and lateral views. The radiographs were obtained at 2 weeks (**a** and **e**), 4 weeks (**b** and **f**), 6 weeks (**c** and **g**), and 8 weeks (**d** and **h**) after surgery. At all time points, there was no bridging callus formation at the fracture site

Four weeks after fracture, 86% of the control group fractures healed, and the union rate was 100% at 6 weeks and 8 weeks after fracture. In contrast, bridging calluses were not seen in the fractures of the non-union group model at 8 weeks after surgery, with the majority classified as atrophic non-union based on their radiographical appearance (Figs. 2, 3). There were significant differences between the non-union group and the control group after 4 and 6 weeks (*P* < 0.05), but there were no significant differences after 2 weeks.

The fractures of the control group formed abundant calluses around the fracture sites at 2 weeks (Fig. 2a and d). Most of the fractured bones formed bridging calluses between both ends, and the cortical gaps disappeared 4 weeks after fracture (Fig. 2b and e). At 6 weeks after fracture, bone remodeling began (Fig. 2c and f).

In the other group, the fractures did not have much callus formation at either end of the fracture sites. Some calluses did form along the bone but away from the fracture ends and did not form bridging calluses at the fracture site (Fig. 3c and d). The ends of the fractured bone were resorbed and became round. Non-union was established at 8 weeks after fracture (Fig. 3h).

### Micro-CT

**Fig. 4:**
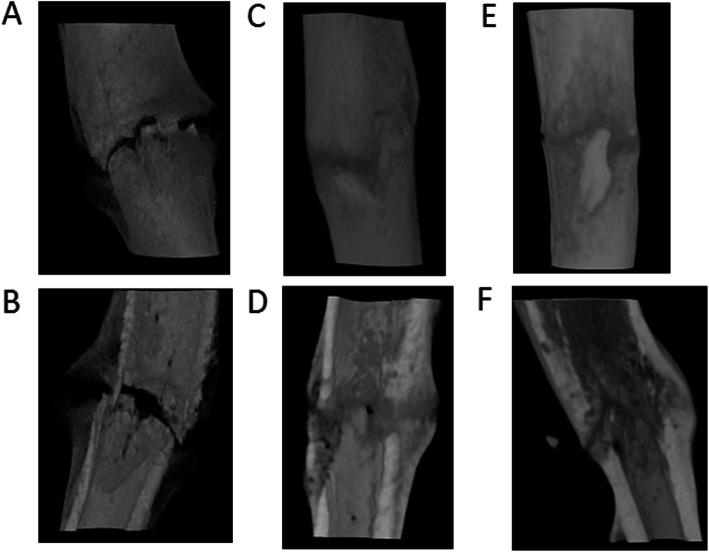
Three-dimensional micro-CT reconstructed images of the control group. The images were obtained at 2 weeks (**a**), 4 weeks (**c**), and 6 weeks (**e**) after surgery and these were viewed by cutting through the long axis of each tibia in the coronal planes (**b**, **d**, and **f**). The fracture gap at 4 and 6 weeks had disappeared and was bridged by new bone formation (**e** and **f**)

**Fig. 5:**
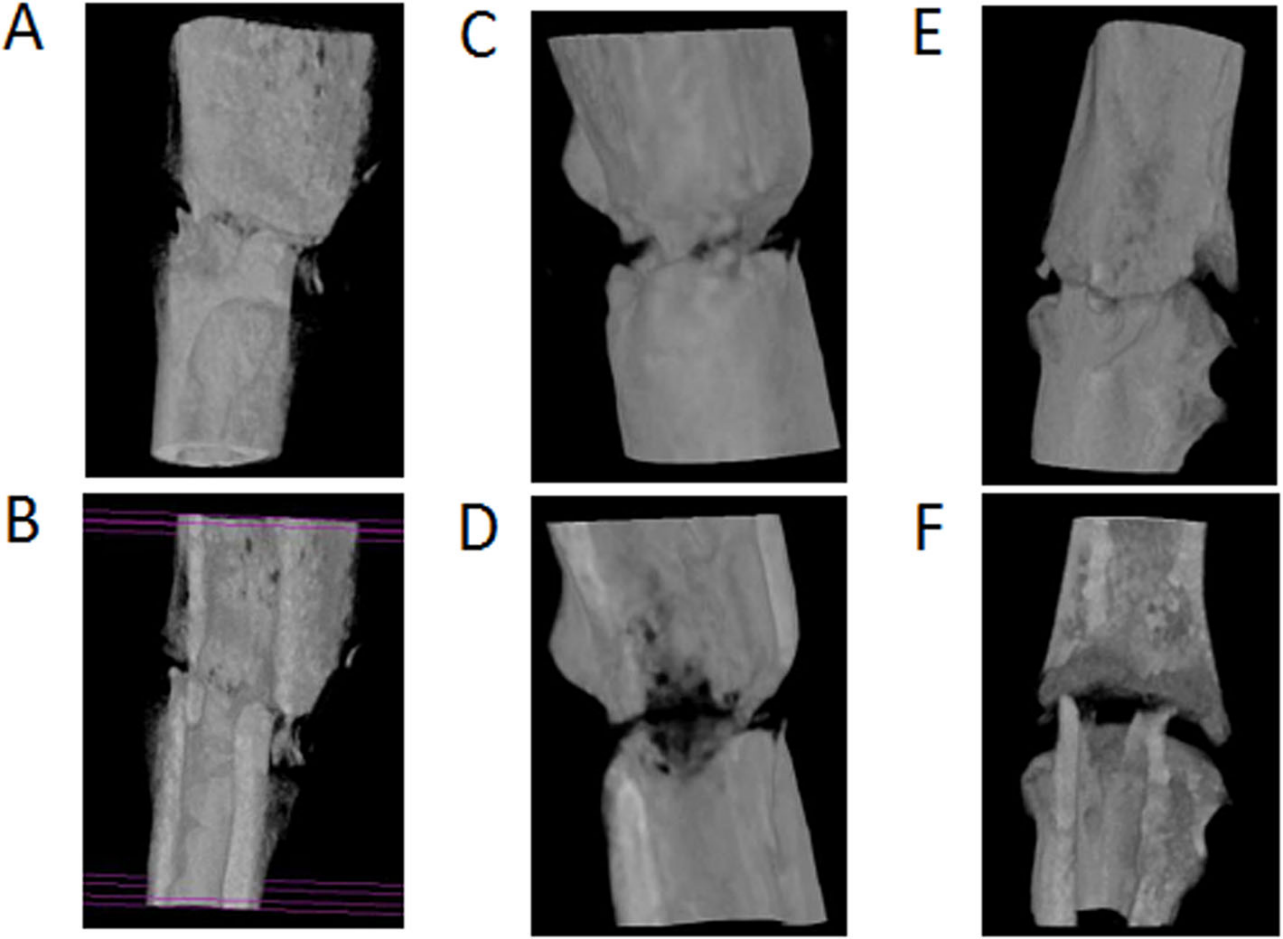
Three-dimensional micro-CT reconstructed images of the non-union group. The images were obtained at 2 weeks (**a**), 4 weeks (**c**), and 6 weeks (**e**) after surgery and these were viewed by cutting through the long axis of each tibia in the coronal planes (**b**, **d**, and **f**). The non-union group tibia persisted as fracture gaps throughout all periods (**b**, **d**, and **f**). As time went on, bone absorption widened the fracture gap

We obtained three-dimensional micro-CT reconstructed images at the same points with the radiographs taken at 2, 4, 6, and 8 weeks after fracture. Typical examples are shown in Figs. 3 and 4. The fracture gap of the control group’s tibia formed abundant calluses at the fracture end and was not bridged completely at 2 weeks (Fig. 4a and b), but the fracture gap had disappeared at 4 weeks and was bridged by new bone formation (Fig. 4c and d). At 6 weeks, the callus had been reconstructed, and its density was similar to that of the cortex. The non-union of the fracture in the non-union group had persistent fracture gaps throughout all periods. Although a small number of calluses formed, they failed to cross the fracture site to form a bone bridge (Fig. 5a-f).

### Histology

**Fig. 6:**
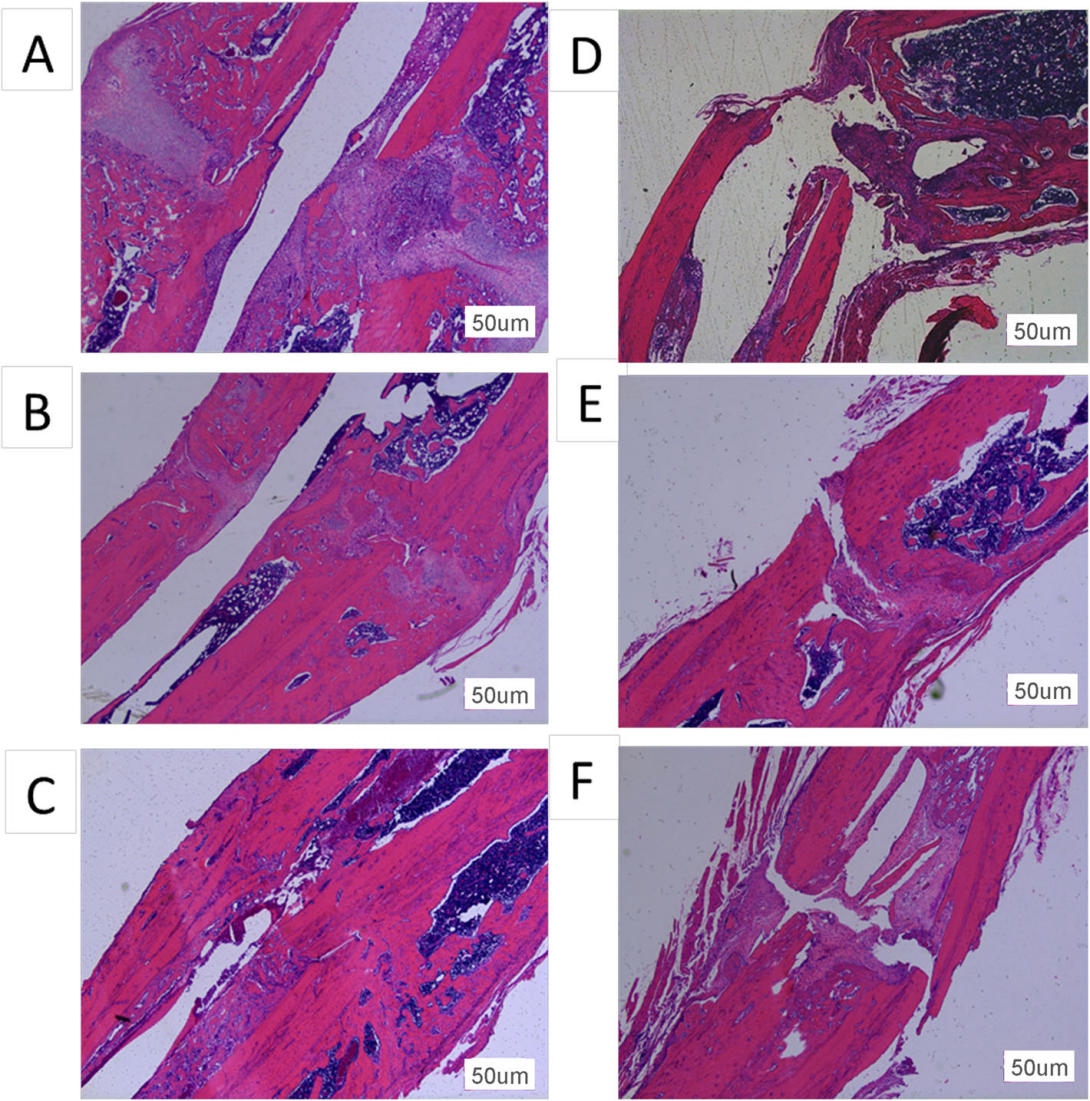
Histological appearance with hematoxylin and eosin staining of the control group models (**a**–**c**) and non-union group models (**d**–**f**) at 2, 4, and 8 weeks. At 2 weeks after fracture, the control group models showed intramembranous ossification and endochondral ossification at the fracture site (**a**). However, there was no ossification on the site of periosteal cauterization in the non-union subjects (**d**). The fracture of the control group united at 4 weeks after surgery (**b**), while the non-union group models still had a large gap (**e**). At 8 weeks, the bridging callus in the control group had remodeled (**c**). In the non-union group, the gap was obvious, and the cortical bone began to be absorbed (**f**). Original magnification ×50

At 2 weeks after fracture, the control group model rats showed intramembranous ossification in the periosteal tissue and endochondral ossification at the fracture site (Fig. 6a). They formed a thick callus consisting of chondrocytes and newly formed trabecular bone, and the two calluses on each side of the fracture nearly united. The gap between the endochondral ossification areas was filled with mesenchymal cells. At the same time, the non-union group exhibited no chondrocytes or endochondral ossification on the site of periosteal cauterization (Fig. 6d). At 4 weeks after fracture, the callus at the fracture end of the control group had united (Fig. 6b). The bone of the fracture end was covered with newly formed trabecular bone and achieved bony union. In contrast, an obvious gap persisted between the surfaces of woven bone in the non-union group at 4 weeks (Fig. 6e). At 8 weeks, the united bone at the fracture end of the control group had remodeled (Fig. 6c). The fracture gap at the original cortical bone interface was bridged by newly formed woven bone, and the border between the cortical bone and newly formed woven bone could hardly be distinguished. In comparison, at 8 weeks in the non-union model, cortical bone resorption was seen at the fracture ends, and the cortical gap was more obvious than before (Fig. 6f). Some non-organized fibrous tissues were seen near the fracture gap. This is consistent with the histological finding of atrophic non-union.

## Discussion

Non-union is a common complication in fracture patients, it is quite difficult to develop a non-union model reproducibly. The appropriate animal models are most important to the studies of the mechanisms of non-union formation [10, 17, 18]. In recent years, many people use rats to make non-union models [10–13, 17]. Reichert et al. [19] reported that rat models are suitable for evaluating the healing process of atrophic non-unions. The tibia of 8-week-old rats is suitable in thickness and convenient for operation and Kirschner wire fixation. Through our pre-experiment, the results show that the medullary cavity of the distal tibia can be matched with 0.8 mm Kirschner wire. The 0.8-mm Kirschner wire can be inserted into the distal tibia near the articular surface and fixed firmly, so 8-week-old rats to make the non-union model were chosen.

A good non-union model should have the following characteristics: reliable, reproducible, simple, easy to operate, and as little physiological stimulation as possible to the body [20]. The early models of non-union tended to be large bone defect models [4, 9]. For technical reasons, osteogenetic defect resection is not suitable for non-unions of femur and tibia [1, 19, 21] because these non-unions cannot adequately simulate the biological environment of a clinical non-union. Some people use placing spacers at the fracture ends to make the non-union model [22]. Although this may improve the success rate, this method’s non-union model can neither imitate a fracture non-union’s biological environment nor be used for rigorous experimental research. Clinical and experimental results indicate that the destruction of the periosteal at the fracture site [6, 8] and instability of the fracture fragments [3, 4] will lead to non-union. Kokubu et al. successfully made a rat model of femoral non-union by using this method [11]. Volpon [23] demonstrated the effectiveness of this method on the radius of a dog. Therefore, we chose the method of periosteum cauterization to make the non-union model.

Currently, most of the rat models mentioned in the literature are femoral non-union models [10–13]. Because of the thick muscle tissues around the femurs, it is difficult to make the model or do the treatment to promote fracture healing. Reed et al. [17] reported one kind of tibial non-union model in rats that can be established by external fixation. This model is not easy to make either. We chose rat tibia to facilitate the establishment of the model and facilitate fracture healing intervention, such as low-frequency pulse, stem cell injection, and other experimental interventions.

Kokubu et al. [11] made a closed fracture model using the technique of Bonnarens and Einhorn by fixing the femur fracture with a 1.25-mm Kirschner wire. A criticism of this study is that the closed fracture model they used as a comparison does not represent a valid sham control. Hietaniemi [10] made a femoral non-union rat model by open osteotomy, Kirschner wire fixation, and cauterization of the periosteum of the fracture sites. We compared these two methods and thought that open osteotomy might keep a better consistency between the control group and the non-union group. This would be better for a controlled study because the non-union group needs to open and expose the periosteum at the fracture end. In Kikuyu’s study, it was found that open osteotomy did not affect fracture healing compared with the closed fracture model. Therefore, we used Hietaniemi’s method to burn the periosteum of the fracture end after open osteotomy of the tibia and then fixed it with a Kirschner wire 0.8 mm in diameter. Using an identical diameter Kirschner wire and the same open osteotomy technique in both the non-union group and control group, we attempted to eliminate the effects of the differences in the mechanical environment on fracture healing.

The periosteum is important for the recovery of fractures [24]. If the soft tissue around the fracture segment is destroyed because of the surgical approach or if the periosteum is widely peeled, it will impair the blood supply of fracture ends and interfere with the normal healing process [5, 6]. These findings [5, 6, 24] showed that blood supply at the fracture site is essential for normal healing and that periosteal destruction will interfere with the blood supply around the fracture site. In our study, we disrupted 2 mm of the periosteum on each side of the fracture site circumferentially. We propose that this interfered with normal fracture healing by reducing periosteal blood supply at the fracture site. Our experiment found that cauterization of the periosteum clearly impaired the normal process of bone healing. We attempted to standardize the region of cauterization and perform a similar soft tissue dissection during osteotomy in each animal to avoid the effect of manipulation variables on the healing of non-union fracture models.

At 2 weeks after fracture, there was already a clear histological difference between the control group and the non-union group. The control group model rats showed obvious intramembranous ossification in the periosteal tissue and endochondral ossification at the fracture site. Four weeks after fracture, the control group’s healing rate was 86%, as shown on the X-ray film, but no subject in the non-union group healed. Micro-CT and histological results showed that the fracture gap had disappeared and was bridged by new bone in the control group. All the control group models healed at 6 weeks, but none of the non-union group models healed. Eight weeks after the operation, there was a clear difference between these two groups. The calluses at the fracture end of the control group had united. The bone at the fracture ends was covered with newly formed trabecular bone, completing the reconstruction of the calluses. In comparison, in the non-union group, the cortical bone resorption made the fracture ends round, and the cortical gap was more obvious than before. Fibrous tissues were seen between the fracture ends. The healing rate was zero for the non-union group throughout the study. The absence of reparative chondrocytes suggests that the fracture healing process was quiescent. This may result from a lack of stimulation of stem cells to form fibroblasts, chondroblasts, or osteoblasts. Marsh [25] has suggested that non-unions should be defined in the cessation of the intramembranous healing response but not the arbitrary terms of duration. At 8 weeks, the non-union model in our experiment appeared atrophic radiographically and histologically, indicating that a fracture non-union had been established.

There are a few limitations in this study. First some people thought that the shape of tibia is irregular. The triangular configuration and the bowed longitudinal axis afford a more sophisticated design of the implants. However, Kirschner wire fixation does not need to span the full length of the tibia, so the fixation is not so difficult as expected. Second may be in the method of development of the fracture non-union. An argument was that typically non-union in humans result from either poor fracture fixation or blood supply disruption of fracture ends, not both of them as we have used. However, previous studies have found that the fracture healing ability of rats is very strong; Kirschner wire fixation of tibial fracture can achieve 100% healing in 4 weeks [14]. Therefore, this model does not prevent us from understanding the non-union caused by the lack of blood supply at the fracture end of periosteal injury.

## Conclusions

We have developed a reproducible atrophic non-union model in rat tibia with atrophic radiographic and histologic appearance compared with the control group model. It is technically simple and easily reproducible. The non-union model showed obvious differences between the physiological healing found in the control group, and the impaired healing led to an atrophic non-union. The non-union was characterized by the cessation of both intramembranous and endochondral ossification adjacent to the fracture site. In conclusion, this method represents a reproducible model to create atrophic non-unions. This model provides a new option for studying the basic mechanisms of healing and evaluating new therapies for bone regeneration and treatment of non-union fractures.

## Data Availability

All data generated or analyzed during this study are included in this published article.

## Abbreviation

ROI: The region of interest
